# Lactated Ringer’s solution versus saline fluid resuscitation for reducing progression to moderate-to-severe acute pancreatitis: a systematic review and meta-analysis

**DOI:** 10.1097/JS9.0000000000002330

**Published:** 2025-03-14

**Authors:** Tang Zhao, Zhiqiang Kang, Qiu Zhang, Feng Pu, Yun Zhang, Wenqing Yin, Hongji Yang, Yu Zhou, Shikai Zhu

**Affiliations:** a Department of Hepatobiliary surgery, The Affiliated Hospital of Southwest Medical University, Lu Zhou, China; b Organ Transplant Center, Sichuan Provincial People’s Hospital, School of Medicine, University of Electronic Science and Technology of China, Chengdu, China; c Sichuan Provincial KeyLaboratory for Clinical Immunology Translational Medicine, Sichuan Provincial People’s Hospital, School of Medicine, University of Electronic Science and Technology of China,Chengdu, China; d Department of Kidney Medicine, Cleveland Clinic Fairview Hospital, Cleveland, Ohio, USA; e Sichuan Provincial Key Laboratory for Human Disease Gene Study, Sichuan Provincial People’s Hospital, School of Medicine, University of Electronic Science and Technology of China, Chengdu, China

**Keywords:** acute pancreatitis, fluid resuscitation, lactate Ringer’s, meta-analysis, normal saline

## Abstract

**Background:**

Fluid resuscitation represents a pivotal early therapeutic intervention in the management of acute pancreatitis (AP), yet a consensus on the optimal fluid type remains elusive. The present study endeavors to elucidate the differential effects of lactated Ringer’s solution (LR) and normal saline (NS) in the initial treatment of AP.

**Methods:**

A comprehensive literature search was conducted through the PubMed, EMBASE, and Cochrane Central Register of Controlled Trials (CENTRAL) databases, spanning from inception until July 2024. The primary outcome of interest was the likelihood of developing moderate-to-severe AP.

**Results:**

This meta-analysis synthesized evidence from six randomized controlled trials (RCTs) and four observational studies, involving a total of 1500 AP patients. Patients were stratified into two groups based on the administered fluid: LR (n = 689) and NS (n = 811). Our findings revealed that, compared to the NS group, patients in the LR group demonstrated a significantly lower risk of moderate-to-severe AP (OR 0.48; 95%Cl 0.34 to 0.67; *P* < 0.001; *I*^2^ = 0%), a shorter hospital stay (MD = −0.74, 95% confidence interval [CI] −1.20 to −0.28, *P* = 0.001; *I*^2^ = 0%), and a reduced intensive care unit (ICU) admission rate [relative risk (RR) = 0.42, 95% CI 0.20–0.89, *P* = 0.02; *I*^2^ = 0%]. Moreover, the LR group also showed a lower incidence of local complications (RR = 0.58, 95% CI 0.34–0.98, *P* = 0.04). Conversely, no statistically significant differences were observed between the two groups in terms of mortality, organ failure rates, Fluid administered 24 h, systemic inflammatory response syndrome (SIRS).

**Conclusions:**

Our analysis underscores the superior efficacy of LR solution in comparison to NS. It provides compelling evidence of LR’s ability to significantly mitigate the onset of moderate to severe pancreatitis. Additionally, our findings reveal that LR is associated with a reduced need for ICU admissions, a lower incidence of local complications, and a shorter overall hospital stay, thereby offering a more favorable clinical outcome. However, no notable differences were discerned in other complications. Subgroup analyses further suggest LR’s potential to curb pancreatic necrosis and other indices, albeit these findings necessitate corroboration through extensive experimentation.

**HIGHLIGHTS**

- This study analyzed 10 studies, including 6 randomized controlled trials and 4 observational studies, with a total of 1,500 patients, ensuring reliable and robust results.
- Rigorous methods were used to review and combine data, reducing bias and increasing reliability. The low heterogeneity across all primary outcomes (*I*^2^ = 0%) highlights the consistency of the findings.
- Compared to previous studies, this research conducted detailed subgroup analyses, identifying differences in treatment effects under various conditions and providing more precise clinical guidance.
- The results demonstrate that lactated Ringer’s solution may be more effective than normal saline in reducing the incidence of moderate to severe pancreatitis, shortening hospital stays, lowering ICU admission rates, and decreasing local complications, offering valuable guidance for fluid resuscitation strategies in acute pancreatitis.

## Introduction

Acute pancreatitis (AP), a pancreatic inflammatory disorder, is characterized by a constellation of clinical manifestations, including vomiting, nausea, upper abdominal or left upper quadrant discomfort, and mild to moderate fever^[^^1]^. Over the past few years, the annual incidence of this condition has escalated by 3.07%^[^^2]^. Notably, while the majority of AP cases present as mild, 20% of patients, albeit an exceptional proportion, develop moderate to severe pancreatitis accompanied by organ failure^[^3-6^]^. The overall mortality rate associated with AP approximates 2–5%, imposing a substantial psychological, physiological, and financial toll on patients due to its intricate and variable clinical course^[^7,8^]^

Given the absence of approved specific therapeutic agents to halt the progression of AP, prompt and effective management is paramount^[^9,10^]^. Intravenous fluid resuscitation serves as the cornerstone of initial management, aimed at preventing hypovolemia and organ hypoperfusion^[^^11]^. However, despite some guidelines advocating for aggressive fluid resuscitation, a consensus regarding optimal fluid resuscitation protocols, particularly the choice of fluid, remains elusive^[^12,13^]^.

Among the available fluid options, crystalloids are generally preferred over colloids, with normal saline (NS) being a commonly utilized fluid in AP management^[^^14]^. Nevertheless, emerging evidence indicates that lactated Ringer’s solution (LR) may surpass NS in mitigating systemic inflammation and improving outcomes in AP patients^[^15-17^]^. Furthermore, NS infusion has been implicated in inducing hyperchloremic acidosis^[^^18]^. A recent single-center investigation revealed that high-volume LR administration may reduce the necessity for intensive care and abbreviate hospital stays in AP cases^[^^19]^. This notion is corroborated by several systematic reviews and meta-analyses, which have compared LR and NS in fluid resuscitation for AP^[^20-22^]^.

Global endeavors by individual researchers, including our own experiences, underscore the need for further validation of LR’s superiority over NS in AP care^[^23,24^]^. Multiple meta-analyses have attempted to consolidate the current evidence base on this topic^[^20,22,25-29^]^. However, given the publication of additional trials subsequent to the last search conducted for these reviews, a more comprehensive analysis is warranted to provide definitive insights^[^15,30^]^. The challenges posed by small sample sizes, low prevalence of moderate-to-severe complications, and scarcity of reports on systemic sequelae hinder the derivation of robust conclusions regarding the benefits of LR. Thus, the present meta-analysis endeavors to consolidate and appraise the latest relevant data, investigating the disparities between LR and NS in the treatment of AP and determining the optimal fluid resuscitation strategy that most favorably enhances AP outcomes.

## Method

### Research question and primary outcome

Our research question was formulated employing the Population, Intervention, Comparison and Outcome (PICO) framework, focusing on fluid resuscitation with LR solution and NS hydration as the intervention. The primary outcome assessed was the occurrence of moderately severe to severe pancreatitis, adhering to the Revised Atlanta Classification criteria^[^^31]^. Secondary outcomes encompassed hospitalization duration, mortality rates, and the onset of systemic inflammatory response syndrome (SIRS). Adhering to rigorous methodology, the study protocol was prospectively registered on PROSPERO and its reporting aligned with PRISMA and AMSTAR guidelines for systematic reviews and meta-analyses, ensuring transparency and quality^[^32,33^]^.

### Literature Search Strategy

**Figure 1.**
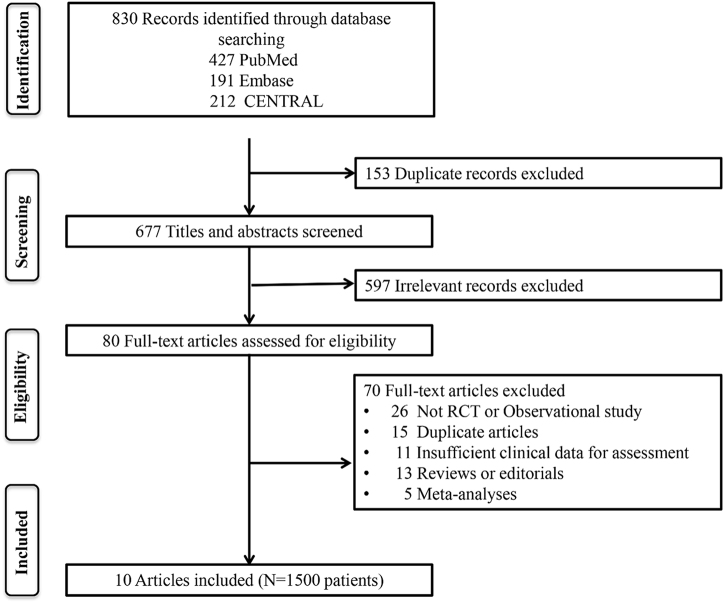
Study selection flow diagram following PRISMA guidelines for evidence synthesis.

A systematic literature search was independently conducted by two reviewers across PubMed, Embase, and the Cochrane Library to retrieve all pertinent original studies. Key search terms employed included “Ringer’s solution”, “Lactated Ringer’s”, “resuscitation”, “fluid therapy,” “hydration”, “hemodilution,” “normal saline,” and variations of “pancreatitis” (e.g., “pancreatic,” “pancreas”, “acute pancreatitis”). This exhaustive search was finalized in July 2024, ensuring inclusivity of the most recent publications. We conducted a comprehensive literature search in the PubMed, EMBASE, and Cochrane CENTRAL databases for studies published between 1 January 1999, and 1 July 2024. This initial search identified 830 articles. In the first screening phase, 153 duplicate articles were excluded. During the second phase, 677 articles that were unrelated to the research topic were removed. Subsequently, two independent reviewers assessed the remaining articles in detail, applying predefined inclusion criteria (such as randomized controlled trials, cohort studies, fluid resuscitation for AP patients, and complete data) and exclusion criteria (such as case reports and data incompleteness). After this thorough evaluation, studies were excluded due to factors such as small sample size and inadequate study design. In the end, 10 studies were selected for further analysis, comprising six randomized controlled trials and 4 observational studies.^[^15-17,19,30,34-38^]^ Furthermore, a manual search of reference lists from relevant articles was undertaken to identify any potentially overlooked studies, thereby augmenting the comprehensiveness of our literature review (Fig. 1).

### Study definitions

Patients with acute pancreatitis (AP) should meet the Atlanta Diagnostic Criteria,^[^^39]^specifically requiring the presence of at least two of the following three criteria: acute onset of epigastric pain, serum lipase or amylase levels at least three times greater than the upper limit of normal, and imaging findings consistent with typical manifestations of AP, The diagnosis of SIRS requires meeting at least two of the following four criteria: (1) temperature >38°C or <36°C, (2) heart rate >90 beats per minute, (3) respiratory rate >20 breaths per minute or PaCO_2_ < 32 mmHg, (4) white blood cell count <4000 cells/mm^3^ or > 12 000 cells/mm^3^ or >10% bands.

Mild acute pancreatitis (MSAP) is characterized by transient organ failure lasting less than 48 hours and/or local or systemic complications without persistent organ failure. In contrast, severe acute pancreatitis (SAP) is defined by persistent organ failure lasting more than 48 hours. Organ failure is assessed using the modified Marshall scoring system^[^^40]^. Local complications include pancreatic necrosis, acute peripancreatic fluid collection, pseudocyst formation, and walled-off necrosis.

### Inclusion and exclusion criteria

The inclusion criteria for study selection were as follows: (1) randomized controlled trials (RCTs) or observational studies were considered; (2) participants were adults aged 18 years or older with AP; (3) studies directly comparing LR and NS in the treatment of AP; (4) reporting at least one of the following outcomes: odds of moderately severe AP, intensive care unit (ICU) admission, mortality, organ failure, hospital length of stay, local complications, or development of SIRS. Conversely, studies were excluded if they: (1) were published as conference abstracts, case reports, or narrative reviews; (2) were not written in English; (3) had data that could not be extracted for analysis.

### Data extraction and quality assessment

Data extraction from each study was conducted independently by two reviewers, with any discrepancies resolved through consultation with a third senior reviewer. The extracted information from each study was as follows: author details, publication year, country, study design, patient demographics (including age and sex), etiology, specifics of fluid therapy (type, administration protocol, and volume), and outcomes comparing LR and NS (Table 1). To address the diverse nature of study designs (RCTs and observational studies), we employed tailored tools for bias assessment: the Cochrane Collaborative Risk of Bias Tool for RCTs and the Newcastle-Ottawa Scale (NOS) for observational studies^[^41,42^]^. The quality of evidence across all outcomes was appraised using the Grading of Recommendations Assessment, Development, and Evaluation (GRADE) framework^[^^43]^. Additionally, publication bias was qualitatively assessed through funnel plot visualization and quantitatively evaluated with Egger’s regression analysis. In cases of suspected publication bias, the “trim-and-fill” method was utilized to gauge potential alterations in effect sizes.

**Table 1:** Basic characteristics of included studies

												Etiology						Fluid in the first 24 h (mL)		
				Patient		Age		Gender (male)		BMI		Gallstone		Alcohol		Other		LR	NS	Fluid administration protocol
Author	Year	Country	Study type	LR	NS	LR	NS	LR	NS	LR	NS	LR	NS	LR	NS	LR	NS			
Wu	2011	USA	RCT	19	21	50(40–73)	54(40–60)	11	7	25	27	8(19)	10 (21)	2 (19)	4 (21)	7 (19)	6 (21)	7200(4300–11 000)	5600(3500–7900)	20 mL/kg bolus for 30 min, 3 mL/kg/h for 8–12 h, after either 20 mL/kg bolus and 3 mL/kg/h or no bolus and 1.5 mL/ kg
Aboelsoud	2016	Israel	R	68	130	63(52–74)	56(44–72)	51										3400	3600	
Lipinski	2015	Poland	R	40	63	49.2 ± 18.0	52.5 ± 17.6	30	38	27.6 ± 5.0	26.3 ± 5.4							4929.57 ± 1265.60	5374.17 ± 768.82	20 mL/kg within 30 min followed by 3 mL/kg/h; BUN controlled
Choosakul	2018	Thailand	RCT	23	24	54.78 ± 20.42	48.33 ± 13.56	12	17			13 (23)	15(24)	8(23)	7 (24)	2 (23)	2 (24)	2160(1704–3200)	2400(2180–3500)	15 mL/kg for 60 min and 1.2 mL/kg/h 10 mL/kg for 60 min and1 mL/ kg/h; 1000 mL 10% dextrose
deMadaria	2018	Spain	RCT	19	21	63.8 ± 19.1	61.4 ± 15.5	8	11	25.2	27.7	14 (19)	15 (21)	0 (19)	4 (21)	5 (19)	2 (21)			
Farrell	2023	USA	RCT	38	38	12.4(10.0–15.0)	14.6(9.5–16.4)	20	13	74.8	92.3	7(38)	13(38)		19(38)		17(38)	3162(2203–3620)	3079(2780–3450)	
Lee	2020	USA	RCT	61	60	42.3 ± 14.0	43.5 ± 14.2	30	33			33(61)	34(60)	17(61)	13(60)	11(61)	13(60)			10 mL/ kg in the first hour; 1.5 mL/kg/h; 1000 mL 5% dextrose
Karki	2022	Nepal	RCT	26	25	41.3 ± 14.2		25	1									3412.97 ± 1606.87	2943.32 ± 1289.14	1000 mL in the first hour; 3 mL/ kg
Kayhan	2021	Turkey	RP	67	65	54.6 ± 17.9	56.3 ± 17.2	23	21			51/67	45/65	4/67	5/65	12/67	15/65			1000 mL; 5% glucose solution (1000–1500 mL); a multielectrolyte solution (500–100 mL)
Lee	2023	Multi-center	RP	328	364	48.2 ± 19.3	51.7 ± 18.9					146(328)	203(364)	47(328)	42(364)	135(328)	119(364)			

### Risk of bias

**Figure 2.**
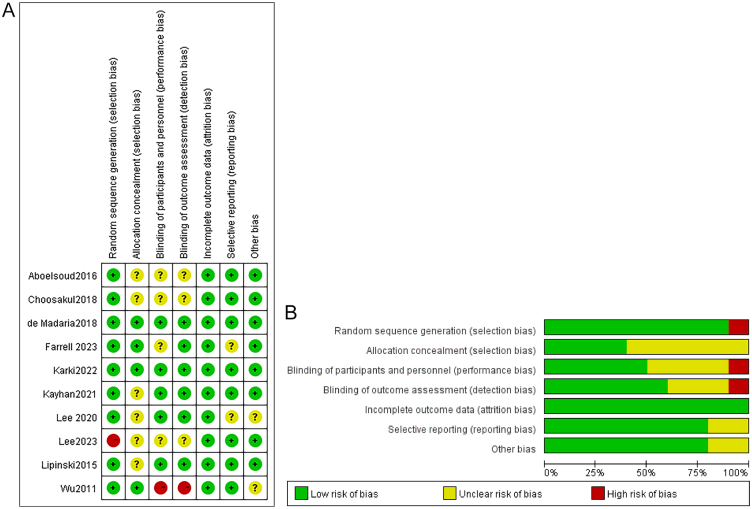
The quality assessment of RCTs. (A) risk of bias summary: summarized authors’ judgments about each risk of bias item for all included studies; (B) Risk of bias graph: sum up authors’ judgments about every risk of bias item which were presented as percentages across all included studies.

The risk of bias in the included trials was independently assessed by two researchers utilizing the Cochrane Collaboration’s Bias Risk Assessment Tool within the Review Manager (version 5.3) software^[^^44]^. This evaluation encompassed five critical domains: randomization process, deviations from intended interventions, missing outcome data, measurement of outcomes, and reporting of results^[^^45]^ (Fig. 2A and B). To ensure rigor and transparency, we adhered to the PRISMA (Preferred Reporting Items for Systematic Reviews and Meta-Analyses) guidelines^[^^32]^. For cohort studies, the Newcastle-Ottawa Quality Assessment Scale (NOS) was employed to gauge quality, focusing on the selection of study populations, comparability of groups, and analysis of outcomes in relation to the exposure of interest^[^^46]^. In contrast, the Jadad Scoring System (JS) was utilized to evaluate the methodological quality of RCTs, particularly examining aspects of randomization, blinding, and the extent of accounting for all patients in the analysis^[^^47]^.

### Statistical analysis

Statistical analyses were conducted utilizing the Review Manager software (version 5.3; Cochrane Collaboration, Copenhagen, Denmark), with additional sensitivity and funnel plot assessments executed in STATA 18. For dichotomous outcomes, the odds ratio (OR) and relative risk (RR) with 95% confidence intervals (CI) was computed. In instances where studies solely presented median and interquartile range for continuous variables, we employed the method outlined by Wan *et al* to derive mean and standard deviation estimates^[^^48]^. Subsequently, the weighted mean difference (WMD) with 95% CI was calculated for continuous outcomes. A random-effects model was adopted to aggregate the original data, with statistical significance set at *P* < 0.05. Heterogeneity across studies was assessed using *Q* and *I*^2^ statistics; an *I*^2^ value exceeding 50% indicated significant statistical heterogeneity. When data for a specific outcome were reported in at least two studies, subgroup analyses stratified by publication type were conducted. Potential publication bias was visually evaluated through funnel plots. The extracted values used to estimate the mean difference (MD) and its variance included the sample size, median, lower and upper quartiles, and the minimum and maximum values for both groups, when available. To estimate the median and its variance for studies reporting means and standard deviations, the quartiles were transformed to fit a normal distribution, and data analysis was conducted using Revman 5.3. The sampling variance of the medians was calculated using the QE method (“Meta-Analysis of the Difference of Medians,” 2020), and a random effects model was employed to summarize the median differences. Additionally, the Hartung–Knapp adjustment was applied to reduce the likelihood of false positive findings^[^49,50^]^.

## Results

### Incidence of moderate-to-severe pancreatitis

**Figure 3.**
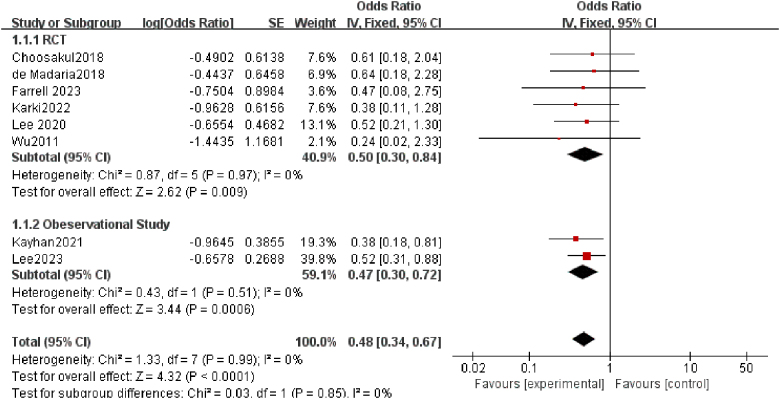
Forest plot of meta-analysis of moderate-to-severe pancreatitis.

Among the studies we included, eight evaluated the clinical outcomes of moderate-to-severe or severe AP based on the revised Atlanta classification^[^^51]^. The pooled analysis revealed that, compared to the NS group, the LR group had a significantly lower risk of developing moderate-to-severe or severe AP (OR = 0.48; 95% CI: 0.34–0.67; *P* < 0.0001; *I*^2^ = 0%). To explore this further, we conducted a subgroup analysis by categorizing the studies into randomized controlled trials (RCTs) and observational studies. The results demonstrated that LR was associated with a significantly lower incidence of moderate-to-severe or severe AP in both RCTs (OR = 0.50; 95% CI: 0.30–0.84; *P* = 0.009; *I*^2^ = 0%) and observational studies (OR = 0.47; 95% CI: 0.30–0.72; *P* = 0.001; *I*^2^ = 0%), with both findings being statistically significant (Fig. 3).

### Length of hospital stay

**Figure 4.**
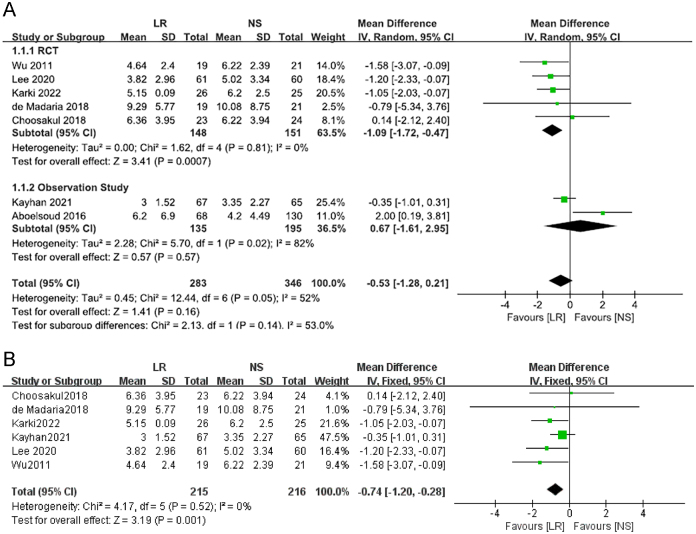
Forest plot of meta-analysis of the total length of hospital stay in the subgroup.(A) RCTs; (B) Observational studies.

The analysis of total hospital stay showed no significant difference between the LR and NS groups (WMD: −0.53 days, 95% CI: −1.28 to 0.21, *P* = 0.16) with moderate heterogeneity (*I*^2^ = 52%) (Fig. 4a). Subgroup analysis by study type revealed a significant reduction in hospital stay for the LR group in RCTs (WMD: −1.09 days, 95% CI: −1.72 to −0.47, *P*<0.001, *I*^2^ = 0%). However, in observational studies, no significant difference was observed (WMD: 0.67 days, 95% CI: −1.61 to 2.95, *P* = 0.57) with high heterogeneity (*I*^2^ = 82%). After analyzing heterogeneity (*I*^2^ > 50%), we excluded the study by Aboelsoud *et al* and performed a reanalysis of the data. The results demonstrated a statistically significant difference (*Z* = 3.19, *P* = 0.001 < 0.05), indicating that LR, compared to NS, effectively reduces hospital stay. Additionally, no evidence of publication bias was observed in the funnel plot. (Fig. 4b).

### ICU admission

**Figure 5.**
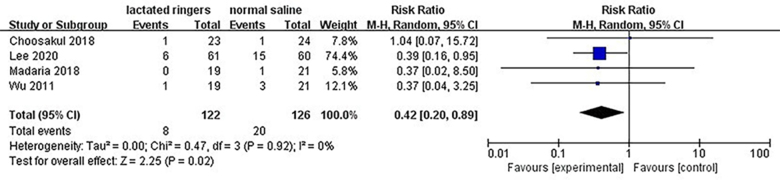
Forest plot of meta-analysis of the rate of ICU admission.

Four RCTs provided detailed insights into the incidence of ICU admission (Fig. 5). Consistently, patients administered with LR exhibited a notably lower rate of ICU admission compared to those receiving NS. Notably, the absence of heterogeneity across these studies (*I*^2^ = 0%) underscores the robustness of this finding, with a pooled RR of 0.42 (95% CI: 0.20 to 0.89; *P* = 0.02) indicating a statistically significant difference favoring LR treatment.

### Incidence of systemic inflammatory response syndrome (SIRS)

**Figure 6.**
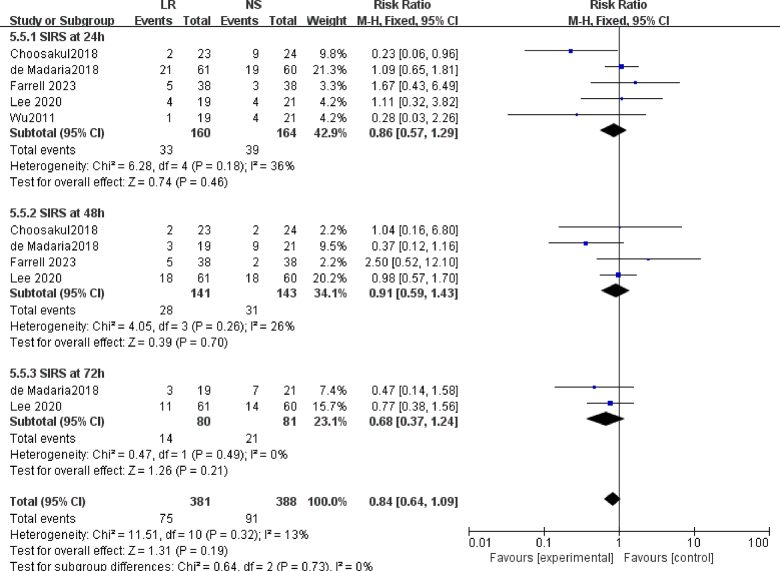
Forest plot of meta-analysis of systemic inflammatory response syndrome at different time after performing fluid therapy.

A subgroup meta-analysis was conducted to evaluate the impact of LR versus NS on the development of SIRS at various time points (Fig. 6). Analysis of five studies (n = 324) revealed no statistically significant difference in the likelihood of SIRS occurrence within 24 hours between patients treated with LR and NS, with a RR of 0.86 (95% CI: 0.57–1.29; *P* = 0.46). Similarly, four studies (n = 284) showed no significant difference in SIRS rates after 48 hours of treatment, with an RR of 0.91 (95% CI: 0.59–1.43). Notably, the heterogeneity among these trials was statistically non-significant (*I*^2^ = 0%; *P* = 0.19). Additionally, two studies failed to detect a significant difference in SIRS probability at 72 hours between the LR and NS groups, yielding an RR of 0.68 (95% CI: 0.37–1.24, *P* = 0.21).

### In-hospital mortality

**Figure 7.**
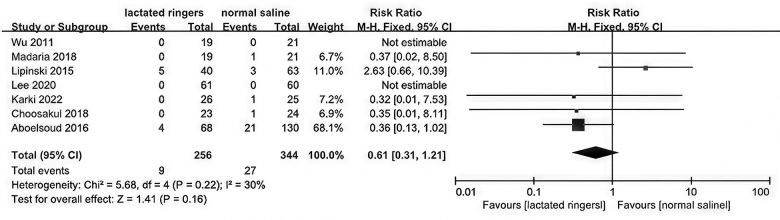
Forest plot of meta-analysis of in-hospital mortality.

Among the 10 included studies, seven contributed data on in-hospital mortality rates. A meticulous meta-analysis of these studies indicated a trend toward reduced mortality among AP patients treated with LR compared to those receiving NS, albeit this difference did not attain statistical significance (*P* = 0.16). Specifically, the RR was estimated to be 0.61, with a 95% CI ranging from 0.31 to 1.21, and a *P*-value of 0.16, indicating a lack of definitive statistical evidence (Fig. 7).

### Complications

**Figure 8.**
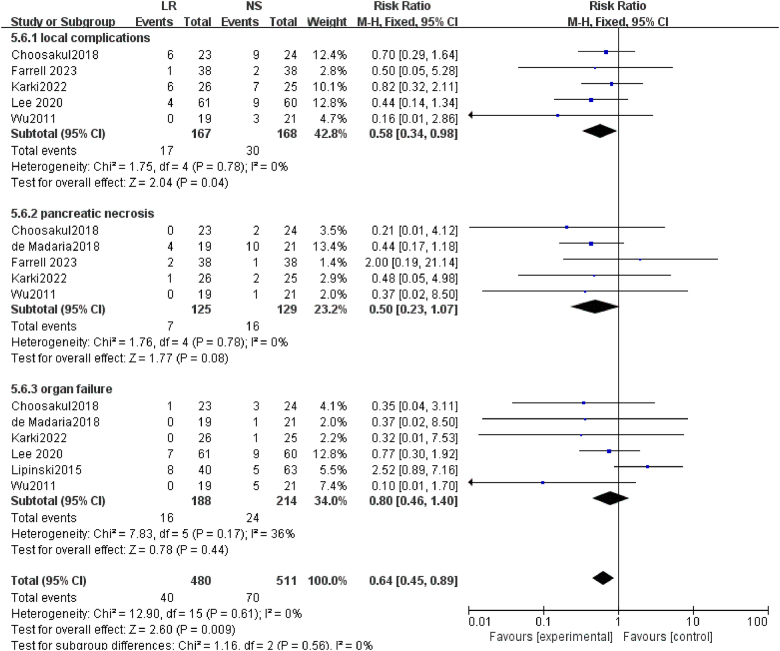
Forest plot of meta-analysis of complications.

In this study, we performed a subgroup analysis focusing on pancreatitis-related complications (Fig. 8). For local complications, data from five studies showed minimal heterogeneity (*I*^2^ = 0%, *P* = 0.78). The pooled analysis demonstrated a significantly reduced risk of local complications in the LR solution group compared to the NS group (RR = 0.58, 95% CI: 0.34–0.98, *P* = 0.04). This finding indicates that the use of LR solution may significantly lower the risk of complications. Regarding pancreatic necrosis, five studies demonstrated a significantly reduced risk in the LR group compared to the NS group (RR = 0.50, 95% CI: 0.23–1.07, *P* = 0.08), with no heterogeneity observed across the studies. For organ failure, six studies were included in the analysis. While there was some variation among the study cohorts, the cumulative effect did not reach statistical significance (RR = 0.80, 95% CI: 0.46–1.40, *P* = 0.44), indicating no clear difference between the LR and NS groups. According to the results of this subgroup analysis, the use of LR solution appears to significantly reduce the risk of local complications compared to NS. While there is a potential trend toward a reduced risk of pancreatic necrosis, the findings for organ failure remain inconclusive and lack statistical significance. The pooled analysis of overall complications suggests that LR solution is more effective than NS in reducing the occurrence of complications, with this difference being statistically significant (*Z* = 2.60, *P* = 0.009). Moreover, no significant heterogeneity was observed across the included studies. However, given the potential limitations related to heterogeneity and sample size, these findings should be interpreted with caution. Further high-quality research is necessary to validate and strengthen these observations.

### Fluid administered at 24 h

**Figure 9.**
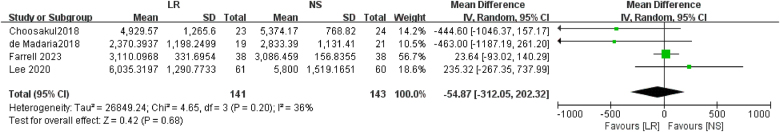
Forest plot of meta-analysis of fluid administered at 24 h.

The analysis of the Fluid administered 24 h revealed no statistically significant disparity between the LR and NS groups, as evidenced by a WMD of −54.87 days with a 95% CI ranging from −312.05 to 202.32 ml (*P* = 0.68), accompanied by moderate heterogeneity (*I*^2^ = 36%). A recent meta-analysis indicated that high-rate, high-flow fluid resuscitation can reduce the incidence of SIRS, shorten hospital stays, and decrease the rate of pancreatic necrosis in AP.^[^^52]^ Previous studies suggest that the benefits of aggressive fluid resuscitation depend on the severity of the condition^[^53,54^]^. The International Pancreas Society and the American Pancreatic Association currently recommend a fluid resuscitation rate of 5–10 mL/kg/h until resuscitation goals are achieved, emphasizing the use of LR solution. During the first 24 hours, the infusion rate should adequately restore circulatory blood volume and urine output. Expert consensus suggests that infusing 2.5–4 liters within 24 hours is sufficient for most patients, but this should be adjusted based on the severity of pancreatitis (Fig. 9).

### Subgroup analysis

Given that our included studies consist of observational research, which may influence the results to some extent, we addressed this by excluding those studies with high heterogeneity. Additionally, we performed subgroup analyses based on various study metrics to assess whether the observed discrepancies originated from the observational studies. This strategy also improves our evaluation of the stability of the research findings.

The subgroup analyses based on study type, specifically randomized controlled trials (RCTs) and observational studies, are presented in Supplementary Digital Content, Table 2 http://links.lww.com/JS9/E5. In the subgroup analysis of ICU admission rates, the admission rate in RCT studies was significantly reduced (OR = 0.02, 95% CI: 0.16–0.87; *P* = 0.0007; *I*^2^ = 0%) (Figure S3, Supplementary Digital Content, http://links.lww.com/JS9/D999). In the subgroup analysis of in-hospital mortality, the OR for RCT studies was 0.33 (95% CI: 0.05–2.16; *P* = 0.25; *I*^2^ = 0%), while the OR for observational studies was 0.91 (95% CI: 0.11–7.68; *P* = 0.93; *I*^2^ = 81%). Neither showed a significant difference (Figure S5, Supplementary Digital Content, http://links.lww.com/JS9/E2). In the analysis of RCTs, the length of hospital stay was significantly reduced, with a WMD of −1.09 days (95% CI: −1.72 to −0.47 days; *P* = 0.0007; *I*^2^ = 0%). Additionally, there was a statistically significant decrease in local complications within the RCT subgroup, with RR of 0.62 (95% CI: 0.36 to 1.05; *P* = 0.08; *I*^2^ = 0%) (Figure S4, Supplementary Digital Content, http://links.lww.com/JS9/D1000). In the subgroup analysis focusing on organ failure, the pooled OR from five randomized controlled trials (RCTs) was 0.43 (95% CI: 0.19–0.97; *P* = 0.04; *I*^2^ = 0%), indicating a statistically significant reduction. In contrast, only one observational study was included in this analysis (Supplementary Digital Content, Figure S6, http://links.lww.com/JS9/E3). In the subgroup analysis of pancreatic necrosis, the OR from five RCT studies was 0.50 (95% CI: 0.23–1.09; *P* = 0.08; *I*^2^ = 0%), while there was only one observational study (Supplementary Digital Content, Figure S7, http://links.lww.com/JS9/E4). We also conducted a subgroup analysis of moderate to severe pancreatitis based on study type. The combined results indicated that, compared to the NS group, the LR group had a lower likelihood of developing moderate to SAP (OR = 0.48; 95% CI: 0.34 to 0.67; *P* < 0.0001; *I*^2^ = 0%). In both RCTs and observational studies, LR was associated with a reduced incidence of moderate to SAP, with RCTs reporting an OR of 0.50 (95% CI: 0.30 to 0.84; *P* = 0.009; *I*^2^ = 0%) and observational studies showing an OR of 0.47 (95% CI: 0.30 to 0.72; *P* < 0.0006; *I*^2^ = 0%).No significant differences from the overall pooled outcomes were observed for other outcomes in the subgroup analyses.

### Heterogeneity

Regarding the heterogeneity among studies investigating moderate to severe pancreatitis, the results demonstrated minimal variation, with *I*^2^ = 0% for the primary outcome and ICU admission rate. Similarly, the subgroup analyses for complications and SIRS did not exhibit substantial heterogeneity. The funnel plot analyses for moderate to severe pancreatitis further confirmed the absence of publication bias and heterogeneity (Supplementary Digital Content, Figure S1, http://links.lww.com/JS9/D998). Additionally, the funnel plot pertaining to mortality indicated no discernible publication bias (Supplementary Digital Content, Figure S2, http://links.lww.com/JS9/E6). While the inclusion of observational studies in the hospitalization rate analysis yielded a relatively high degree of heterogeneity (*I*^2^ = 82%), the RCTs within this cohort displayed low heterogeneity (*I*^2^ = 0%), underscoring the robustness of the randomized controlled trial design in mitigating variations across studies.

## Discussion

AP is a significant contributor to hospitalization globally, associated with considerable morbidity and healthcare costs^[^^55]^. Early aggressive fluid resuscitation is critical in managing patients with confirmed AP, especially during the initial stages of the condition^[^^56]^. Nevertheless, the optimal fluid regimen for resuscitation in AP remains a contentious issue. This comprehensive meta-analysis represents the most recent comparative evaluation of LR and NS fluid resuscitation strategies for AP, revealing a significant association between the type of fluid administered within the initial 24 hours of admission and the severity of the disease. Our findings, corroborated by subgroup analyses, underscore the notable reduction in risks of moderately severe to severe AP when LR is utilized within this critical timeframe. Furthermore, LR administration was associated with a statistically significant decrease in ICU admissions, underscoring its efficacy in mitigating disease progression. Comparatively, resuscitation with LR markedly shortened hospital stays and diminished the incidence of local complications in AP patients, as compared to NS. These results reinforce the pivotal role of LR in the management of AP, emphasizing its importance as a therapeutic intervention^[^8,57^]^.

Hydration and pain management are pivotal elements in the therapeutic strategies for AP^[^8,57,58^]^. Beyond averting pancreatic necrosis, hydration addresses intravascular volume depletion stemming from vomiting, diminished oral intake, fluid sequestration, and diaphoresis^[^^59]^. Colloids of larger molecular weights, such as albumin, heptastich, and dextran, exhibit superior retention within the intravascular compartment, thereby optimizing hemodynamic responses. Although colloids facilitate sustained circulatory flow amidst heightened vascular permeability, their utilization is constrained by potential complications, including coagulopathy, intravascular volume overload, hyper oncotic renal dysfunction, and, albeit rarely, allergic reactions.

Numerous clinical trials comparing the efficacy of LR and NS in AP management have yielded inconclusive results. Consequently, we embarked on a systematic review to evaluate the comparative effectiveness and safety of LR solution as opposed to NS in the treatment of patients with AP. Our analysis demonstrated that LR holds a marginal edge over NS in diminishing the likelihood of moderate to severe manifestations of AP. Moreover, LR exhibited a clear advantage in curtailing hospital stay durations and potentially in reducing the risk of developing local complications. Despite these findings, no statistically significant disparities were observed in critical outcomes such as the occurrence of SIRS at multiple post-resuscitation intervals, in-hospital mortality rates, the incidence of pancreatic necrosis, or the prevalence of organ failure. In the context of surgical interventions, our study did not discern any notable differences in the overall rates of local complications when LR was compared to NS.

Subgroup analyses within our review pointed towards a potential benefit of LR in mitigating pancreatic necrosis relative to NS, a finding that warrants further investigation in future research endeavors. Notably, our data indicate that patients treated with LR experienced a significantly shorter hospital stay when compared to those treated with NS, a result that stands out as statistically significant and clinically meaningful. A recent meta-analysis demonstrated that an aggressive hydration regimen can effectively reduce the length of hospitalization in patients with MAP, supporting our findings^[^^60]^.

Our meta-analysis has yielded significant findings, demonstrating that the administration of LR solution is associated with a notable reduction in the overall incidence of complications, as evidenced by a *P*-value of 0.009, which indicates statistical significance. This suggests that LR solution may be a superior choice in the management of patients, potentially leading to better clinical outcomes. Mortality and irreversible organ failure constitute objective indicators of the effectiveness of AP treatment^[^^36]^. Conversely, organ failure and mortality rates typically do not exceed 5%, posing a challenge for demonstrating treatment-related improvements within the confines of current sample sizes. Despite this, the study’s findings indicate that no significant difference in mortality was observed between the NS and LR groups, both in observational and RCT settings. This aligns with previous meta-analyses, reinforcing the consistency of the findings^[^^22]^. Notably, inconsistencies in mortality reporting across the two observational studies and modest sample sizes in the RCTs, coupled with an inadequate number of reported deaths, hindered the attainment of a substantial effect size. Intriguingly, the study unveiled a potential benefit of LR in reducing the risk of organ failure (RR = 0.55, *P* = 0.12), albeit this trend failed to elucidate the timing of organ failure due to the marked divergence in prognosis between persistent organ failure and transient early organ dysfunction (<48 hours). To furnish clinical practice with a more definitive guide, it is anticipated that future high-quality studies will incorporate detailed recording of organ failure at various time points (both within and beyond 48 hours) and employ larger sample sizes, thereby enhancing the robustness and reliability of the findings.

SIRS stands as a pivotal indicator of the severity of AP. Prior research has underscored the protective role of LR against the onset of SIRS in AP patients. The majority of studies encompassed in this meta-analysis assessed SIRS rates at varied intervals (24, 48, and 72 hours), yet failed to detect statistically significant disparities between patients undergoing LR and those treated with NS. The heterogeneity of this indicator across studies cannot be overlooked, potentially due to varying proportions of patients with SIRS at admission, significant differences in the etiology of pancreatitis, patient age, and fluid volume^[^^22]^. Furthermore, a recent large RCT assessed aggressive fluid resuscitation in patients with AP. Early termination of this trial occurred after the first interim analysis due to fluid overload, highlighting this safety concern as an important factor when selecting a fluid type.^[^^61]^ C-reactive protein (CRP), due to its universal accessibility, cost-effectiveness, and status as a non-specific inflammatory marker, has been thoroughly validated as a reliable predictor of AP severity. Studies have shown that patients treated with LR experienced a significant reduction in CRP levels within 24–48 hours compared to those receiving NS, underscoring the anti-inflammatory capabilities of LR in managing AP^[^16,34^]^. However, the current study did not encompass CRP data analysis, leaving a gap in our understanding of its dynamic changes in the context of different treatment modalities. It is hoped that future research endeavors will delve deeper into CRP dynamics, offering more comprehensive insights into the inflammatory processes underlying AP and the efficacy of various therapeutic interventions.

Over the past 2 years, four meta-analyses and systematic reviews have assessed the efficacy of LR versus NS fluid resuscitation for AP^[^20-22,27^]^. However, a dearth of eligible studies and inadequate sample sizes limited their ability to yield definitive conclusions. Notably, Calderón *et al*’s interpretation of pooled outcomes was potentially biased^[^^27]^, while Osyka *et al*’s work^[^^21]^, incorporating recent studies, suffered from language and publication biases. Lee *et al*’s study^[^^37]^, with a substantially larger sample size, emerged as the first international-scale endeavor in this field. Three primary limitations underscore the need for caution in interpreting these findings: scarcity of research, small sample sizes across studies, and inability to assess publication bias comprehensively. Despite the inherent limitations of our study, the analysis we conducted suggests that LR solution could potentially provide significant benefits over NS in the management of patients with MAP-to-SAP. Specifically, LR may lead to a reduction in the incidence of local complications, shorten the duration of hospital stays, and decrease the need for ICU admissions. These findings highlight the possible advantages of LR in the clinical setting, offering a compelling case for further investigation into its use as a resuscitation fluid in the treatment of AP. Nonetheless, further research with larger, multicenter randomized controlled trials is imperative to validate these findings and explore LR’s impact on SIRS, mortality, ICU admissions, and organ failure. Future investigations should also explore LR’s mechanisms in mitigating pancreatic necrosis and local complications, compare it with alternative fluid resuscitation strategies, and evaluate optimal fluid choices for AP treatment. Ultimately, such endeavors will contribute to refining clinical guidelines and optimizing patient outcomes.

In addition, the WATERLAND trial, which began in 2024, is an international, multicenter, open-label randomized controlled trial designed to comprehensively compare the resuscitation effects of LR solution and NS in patients with AP^[^^62]^. This study aims to validate LR as the optimal resuscitation fluid choice in AP treatment through a large sample design and to investigate its potential efficacy in alleviating moderate to severe AP. These findings further reinforce the conclusions of our meta-analysis, highlighting the significant role of LR in managing moderate to severe cases of AP.

Our subgroup analysis could not completely adjust for all potential confounding factors, which might affect the reliability of the analysis to some extent. However, the subgroup analysis does not impact the main results of the overall analysis. Given that our study included observational research, we aimed to conduct further analysis by dividing the two groups into subgroups to refine the results and reduce the potential bias introduced by observational studies on the overall findings. This approach allows us to verify the robustness of the results to a certain extent and further strengthen the support for our research conclusions. In the course of our research, we have taken into account the potential impact of the type of monitoring employed and the underlying clinical conditions of patients on the outcomes associated with fluid resuscitation in AP. We are aware that these elements could lead to variations in how patients respond to treatment, which in turn could affect the applicability of our results across different settings. To mitigate this, we have performed subgroup analyses designed to evaluate the uniformity of treatment effects when considering diverse monitoring techniques and patient subgroups.

Admittedly, our study may not fully encapsulate the complexity introduced by these factors, which could potentially shade our interpretation of the results. The diversity in monitoring practices across various centers, coupled with the inherent heterogeneity within patient populations, likely played a role in shaping the outcomes we observed. In light of this, we advocate for future investigations to prioritize the standardization of monitoring protocols and to extend their reach to include a broader spectrum of patient demographics. Such an approach will be instrumental in gaining a more nuanced understanding of how these factors influence treatment outcomes.

The kidneys are highly vulnerable during SAP, primarily due to early renal hypoperfusion caused by hypovolemia and persistent inflammation. NS infusion can elevate plasma chloride levels, leading to hyperchloremic metabolic acidosis, which may worsen inflammation and impair renal perfusion. In contrast, Ringer’s acetate, with a chloride concentration closer to that of human plasma (94–111 mmol/L), may mitigate these risks^[^63,64^]^. Critically ill pancreatitis patients often present with homeostatic imbalances and complications like sepsis. While LR is isotonic and closely resembles plasma, its lactate content can cause lactic acidosis with excessive use. Ringer’s acetate, however, can correct lactic acidosis, enhance lactate clearance, and improve microcirculatory function during shock^[^^65]^. In such cases, Ringer’s acetate may be more effective in addressing acid-base imbalances due to its superior metabolic profile. Although our study focused on comparing LR and NS, we acknowledge the potential advantages of Ringer’s acetate in specific conditions like metabolic acidosis and hyperlactatemia. Future studies should explore direct comparisons between Ringer’s acetate and LR solutions.

Our study possesses several strengths: (1) It represents the most up-to-date meta-analysis to date, encompassing the largest number of studies and the highest sample size. We have included a total of 10 studies, comprising 6 Randomized Controlled Trials (RCTs) and 4 observational studies, with subgroup analyses conducted for both RCTs and observational studies across various outcomes; (2) There is evidence of low to moderate heterogeneity among the studies, and no signs of publication bias have been detected; (3) Unlike previous research, we have conducted a more nuanced stratified analysis of key subgroups, allowing us to uncover heterogeneity in treatment effects under different conditions; (4) While previous reviews have primarily focused on outcomes such as hospital stay, SIRS, and complications, our study broadens the scope of analysis to include the incidence of moderate to severe pancreatitis and differences in fluid volume, offering a more comprehensive perspective on this topic, which has been less explored in prior studies.

This study does have some limitations: (1) Not all included studies were Randomized Controlled Trials (RCTs); four were observational studies, which may introduce bias and diminish the credibility of the findings; (2) Individual patient information was not available, preventing us from stratifying the results by the etiology and severity of pancreatitis; (3) Despite detailed fluid administration, there was no standardized fluid administration protocol across studies, including fluid rates, volumes, and the timing of the initiation of resuscitation; (4) We did not analyze CRP levels, an important marker for inflammation. To address the inherent confounding factors in the included observational studies, we conducted subgroup analyses to mitigate their impact. Furthermore, compared to previous research, our results are more robust and reliable due to the increased sample size, providing a stronger foundation for future clinical decision-making. We acknowledge that monitoring methods and patients’ clinical conditions are key factors influencing treatment outcomes and the generalizability of our findings. Advanced monitoring technologies may detect subtle physiological changes more accurately, while standard methods might underestimate these changes, contributing to the heterogeneity observed in our study. Similarly, differences in disease severity, comorbidities, or baseline treatments may also affect outcomes, particularly in observational studies where confounding bias is more likely. Despite our efforts to adjust for these factors, the potential for unmeasured confounders remains. These limitations should be considered when interpreting our results. Future studies should address variations in monitoring methods and include more detailed stratified analyses to further validate and refine our findings. While our study underscores the importance of fluid resuscitation in improving the course of AP, we recognize the limited evidence regarding its impact on key clinical outcomes such as mortality and SIRS. Additionally, fluid therapy is only one of many factors influencing prognosis, with interventions like nutritional support, antibiotic use, and complication management also playing critical roles. We recommend future research adopt multifactorial approaches to evaluate the interplay between fluid resuscitation and other treatments, aiming to further optimize the management of AP.

In conclusion, LR resuscitation is associated with a reduction in moderate-to-severe pancreatitis, lower ICU admission rates, a significant shortening of hospital stays, and a decreased risk of local complications. A recent meta-analysis^[^^60]^ demonstrated that an aggressive hydration regimen can effectively reduce the length of hospital stay in patients with MAP, further supporting our findings. For all other outcomes, including overall mortality, SIRS and organ failure, no significant differences were observed between the two groups. Based on these findings, we recommend LR as the first-line resuscitation therapy for patients with AP.

This study highlights the role of fluid resuscitation strategies in the management of AP; however, their impact on long-term outcomes such as mortality and SIRS warrants further investigation. Future high-quality randomized controlled trials should integrate fluid therapy with other key interventions, including nutritional support and complication management, to comprehensively evaluate treatment efficacy. Larger-scale studies are needed to validate our findings and to clarify the role of LR solution in reducing SIRS incidence, in-hospital mortality, and 24-hour fluid requirements, providing stronger evidence for the precision management of AP.

## Data Availability

The material of this article is original research. All data in this manuscript is available and transparent for readers

## Research registration unique identifying number (UIN)

The protocol for this meta-analysis has been registered with PROSPERO (CRD42024544237) at https://www.crd.york.ac.uk/PROSPERO#recordDetails.
